# Transmembrane serine protease TADG-15 (ST14/Matriptase/MT-SP1): expression and prognostic value in ovarian cancer

**DOI:** 10.1038/sj.bjc.6602320

**Published:** 2004-12-21

**Authors:** H Tanimoto, K Shigemasa, X Tian, L Gu, J B Beard, T Sawasaki, T J O'Brien

**Affiliations:** 1 Department of Gynecology, Higashi-Hiroshima Medical Center, Higashi-Hiroshima, Japan; 2 Department of Obstetrics and Gynecology, Hiroshima University Graduate School of Biomedical Sciences, 1-2-3, Kasumi, Minami-Ku, Hiroshima 734-8551, Japan; 3 Department of Obstetrics and Gynecology, University of Arkansas for Medical Sciences, Little Rock, AR, USA

**Keywords:** TADG-15, ST14, ovarian cancer, immunohistochemistry, semiquantitative PCR, prognosis

## Abstract

Tumour-associated differentially expressed gene-15 (TADG-15/ST14/matriptase/MT-SP1) is a novel member of the transmembrane serine proteases. Previous studies indicated that TADG-15 is overexpressed in ovarian tumours; however, relationships between expression of TADG-15 and clinical characteristics of ovarian cancer remain unclear. The purpose of this study was to examine TADG-15 expression in ovarian cancers and determine any associations with clinicopathological characteristics or patient survival. Immunohistochemical study revealed that TADG-15 was expressed in 50 (56.2%) of 89 ovarian carcinomas, whereas it was not detected in normal ovaries. TADG-15 expression was significantly more common in patients with early stage disease compared with patients with advanced stage diseases (namely, stage I, 24 out of 33: 72.7%; stage II/III/IV, 26 out of 56: 46.4%; *P*=0.0157). Kaplan–Meier survival curves demonstrated that patients with TADG-15-positive tumours have had substantially longer survival (*P*=0.0480). The mean value of relative TADG-15 mRNA expression ratio was significantly higher in stage I tumours than in stage II/III/IV tumours (*P*=0.0053). Increased expression of TADG-15 is frequently detected in early stage cancers, with expression level downregulated during progression of disease. TADG-15 is associated with early stage ovarian cancer and longer patient survival; therefore, it may be a favourable prognostic marker for this malignancy.

Proteases mediate specific proteolysis and contribute to numerous biological processes, including embryo morphogenesis, tissue rearrangement, complement activation, and blood coagulation (Neurath, 1989). It is well documented that tumour progression and invasion require the combined action of many proteolytic enzymes such as metalloproteases and serine proteases (Mignatti *et al*, 1986; Tryggvason *et al*, 1987; Liotta *et al*, 1991; Duffy, 1992). These enzymes mediate the dissolution of extracellular matrix components surrounding tumour cells, catalyse the degradation of intercellular cohesive structures that allows shedding of tumour cells into the extracellular environment, and activate growth and angiogenic factors during tumour progression.

To assess the value of secreted proteases as markers for early tumour detection and as targets for therapeutic intervention, we developed a strategy to detect serine protease genes differentially expressed in ovarian cancer using redundant primers to the amino-acid sequences comprising the conserved catalytic triad domain of the serine protease family (viz. His–Asp–Ser). Using this approach, we have previously reported that several candidates including hepsin, stratum corneum chymotryptic enzyme (SCCE/KLK7), protease M (KLK6), TADG-12, and TADG-14 (KLK8) are abundantly expressed in ovarian cancers (Tanimoto *et al*, 1997, 1999, 2001a; Underwood *et al*, 1999, 2000). In addition, we have identified and cloned a novel serine protease tumour-associated differentially expressed gene-15 (TADG-15/ST14/matriptase/MT-SP1) as reported in 1998 (Tanimoto *et al*, 1998).

TADG-15 is a transmembrane multidomain serine protease that includes two CUB repeats (*c*omplement subcompliments C1r/C1s, *U*egf, and *b*one morphogenetic protein 1), four ligand binding repeats of the low-density-lipoprotein receptor (LDLR) like domain, and a serine protease domain in the extracellular space (Tanimoto *et al*, 2001b). The total length of the cDNA is approximately 3.15 kb, and the nucleotide sequence includes a single open reading frame for a protein of 855 amino acids. The gene sequence was also identified as ST14 through subtractive hybridisation analysis by Zhang *et al* (1998). Takeuchi *et al* (1999) identified a sequence called MT-SP1 from the human prostate cancer cell line PC-3. MT-SP1 is almost 100% identical to the TADG-15 sequence at the nucleotide and amino-acid levels. There is one amino-acid difference at the protein level, which is most likely due to a sequencing error. Lin *et al* (1999) have described the cloning of a matrix-degrading serine protease named matriptase, which also appears to be homologous to TADG-15 with a structure and homology to TADG-15 in the CUB, LDLR-L, and protease domains. Matriptase was initially isolated from breast cancer cells as an 80-kDa enzyme with gelatinolytic activity (Shi *et al*, 1993). Subsequently, Lin *et al* (1999) revised their sequence to include a cytoplasmic domain, a transmembrane domain, and an extracellular domain upstream from the CUB, LDLR-L, and protease domains described for TADG-15. In addition, Kim *et al* (1999) cloned a mouse transmembrane protease named epithin. Epithin and TADG-15 are 84% similar over 855 amino acids, which suggests that TADG-15 may be the human orthologue of this mouse protein.

Recently, several membrane-anchored proteins sharing a number of common structural features including a proteolytic domain, a transmembrane domain, a short cytoplasmic domain, and a variable length stem region containing modular structural domains were described as a new subfamily of serine proteases – the type II transmembrane serine proteases (TTSPs) (Hooper *et al*, 2001). The majority of TTSPs appear to be implicated in tumour growth, progression, and metastasis. TADG-15 (ST14/matriptase/MT-SP1) shares these common structural features of the TTSPs. Analyses of the substrate specificity of TADG-15 (ST14/matriptase/MT-SP1) reveal that the enzyme cleaves and activates the hepatocyte growth factor/scattering factor and urokinase plasminogen activator (Lee *et al*, 2000; Takeuchi *et al*, 2000). These substrate proteins are known to play important roles in tumour development.

In the present study, we tested the hypothesis that TADG-15 may have prognostic value as an ovarian cancer biomarker by investigating the expression of TADG-15 with immunohistochemical techniques in ovarian carcinomas of different stages, grades, and histological types; moreover, we correlated TADG-15 expression with clinicopathological parameters and patients' survival with the use of statistical analyses. In addition, to validate the mRNA expression level, we examined TADG-15 mRNA expression with semiquantitative PCR.

## MATERIALS AND METHODS

### Patients and tissue specimens

Epithelial ovarian cancer specimens were obtained from 89 patients (mean age 51.3 years, range 23–80 years) who had been treated surgically at the Department of Obstetrics and Gynecology, Hiroshima University Hospital (Hiroshima, Japan) between 1988 and 1999. Normal ovaries were obtained from seven patients who underwent surgery for benign gynaecologic disease. Informed consent was obtained from each subject according to institutional guidelines. The primary pathologic diagnoses for the cancer patients were serous adenocarcinoma for 39 patients, mucinous adenocarcinoma for 19 patients, endometrioid adenocarcinoma for 17 patients, and clear cell adenocarcinoma for 14 patients. Clinical staging was determined according to the criteria of the International Federation of Gynecology and Obstetrics (FIGO). Of the 89 patients, 33 patients had stage I disease, 11 had stage II, 41 had stage III, and four had stage IV disease. All patients received radical or optimal cytoreductive surgery as the first step in treatment and underwent complete clinical staging based on the final histological and cytological findings after surgery to exclude the presence of occult metastatic disease. Stage Ia or Ib and grade 1 patients received no further treatment. Most of the patients who had stage Ic, II, III, or IV disease were treated with postoperative platinum-based chemotherapy. A total of 11 patients received paclitaxel as well as platinum agents. In the follow-up care clinic for ovarian cancer patients, the patients were seen every month in the first year of follow-up, every 2 months in the second year, every 3 months in the third year, and every 6 months in the fourth and the fifth year; 5 years or more after initial therapy, annual follow-up care was continued. The mean follow-up period was 53.0 months (range 3.5–169.1 months). All specimens were fixed in 10% neutral-buffered formalin and embedded in paraffin for immunohistochemistry.

For the semiquantitative PCR analysis, fresh surgical specimens from 51 ovarian cancers were available. These materials were obtained immediately following the surgical procedure and were cut in half. One half of each specimen was processed for histological examination to determine the percentage of tumour cells (never lower than 80% of all cells in the sample), while the other half was frozen in liquid nitrogen and stored at −80°C prior to mRNA isolation.

### Immunohistochemistry

Formalin-fixed and paraffin-embedded sections, 4 *μ*m thick, were cut and mounted on aminopropyltriethoxysilane-treated slides. Slides were routinely deparaffinised with xylene and rehydrated with a series of ethanol washes. Nonenzymatic antigen retrieval was performed by processing with use of microwave heat treatment in 0.01 M sodium citrate buffer (pH 6.0). Immunohistochemical staining was performed using the avidin–biotin peroxidase complex technique (Vectastain Elite ABC kit, Vector Laboratories, Burlingame, CA, USA) according to a previously described method with some modifications (Tanimoto *et al*, 2001b). Anti-TADG-15 rabbit polyclonal antibody was generated by immunisation with a combination of 2-poly-lysine-linked multiple Ag peptide derived from the TADG-15 protein sequence as described previously (Tanimoto *et al*, 2001b). The final products were visualised using the 3-amino-9-ethylcarbazole (AEC) substrate system (DAKO Co., Carpinteria, CA, USA). Both positive and negative controls were used for each section. Ovarian carcinoma samples known to show positive TADG-15 staining were used as positive controls according to published literature (Tanimoto *et al*, 2001b). Normal rabbit serum IgG (Vector Laboratories, Burlingame, CA, USA) was used as a negative control in place of the primary antibody. All experiments were duplicated. The percentage of positive tumour cells was scored as follows. When no positive tumour cell stain could be identified or when fewer than 10% of the focally distributed tumour cells were positive, staining was considered negative. When more than 10% of the tumour cells showed positive staining, staining was considered positive. All stained slides were scored independently by two of the authors (KS and XT).

### Semiquantitative PCR

Extraction of mRNA from the tissue specimen and cDNA synthesis were carried out by methods described previously (Shigemasa *et al*, 1997; Tanimoto *et al*, 1997, 2001b). The mRNA was isolated using a RiboSepTM mRNA isolation kit (Becton Dickinson Labware, Bedford, MA, USA). The cDNA was synthesised with 2.0 *μ*g of mRNA by random hexamer priming using the AdvantageTM RT-for-PCR kit (Clontech, Palo Alto, CA, USA).

Expression levels of TADG-15 mRNA were examined by a semiquantitative PCR technique with minor modifications to a previously described method (Shigemasa *et al*, 1997; Tanimoto *et al*, 1997, 2001b). The TADG-15 sense primer sequence was 5′-ATGACAGAGGATTCAGGTAC-3′ and the antisense primer sequence was 5′-GAAGGTGAAGTCATTGAAGA-3′. The internal control was *β*-tubulin. The *β*-tubulin sense primer was 5′-TGCATTGACAACGAGGC-3′, and the antisense primer was 5′-CTGTCTTGACATTGTTG-3′. Reaction mixtures consisted of cDNA derived from 50 ng mRNA, 5 pmol sense and antisense primers, 200 *μ*mol dNTPs, and 0.625 U *Taq* DNA polymerase with reaction buffer (Takara Biochemicals, Kyoto, Japan) in a final volume of 25 *μ*l. A total of 30 cycles of PCR were carried out in a Thermal Cycler (Perkin Elmer, Foster City, CA, USA). Each cycle of PCR included 30 s of denaturation at 94°C, 60 s of annealing at 60°C, and 60 s of extension at 72°C. We confirmed that amplification under these conditions resulted in linear production of each product. PCR products were separated on 2.0% agarose gels, and the density of each PCR product was determined using a Printgraph-Densitograph system (ATTO Co., Tokyo, Japan). In the present study, we used the expression ratio (TADG-15/*β*-tubulin) as measured by a Printgraph-Densitograph system to evaluate gene expression.

### Statistical analysis

For statistical analysis, the *χ*^2^-test of significance and the Fisher's exact test were used to analyse the distribution of TADG-15 expression in the cancer cases according to their clinicopathological characteristics. A Kaplan–Meier survival curve of ovarian cancer patients was prepared according to TADG-15 protein expression status, with differences in overall survival rates determined by the log-rank test. Overall survival time was defined as the period between initial surgery and the time of death. Survival times of patients who were still alive were noted along with the date of the most recent follow-up appointment. For multivariate analyses, we used the Cox proportional hazards model. The unpaired *T*-test was used to assess the differences of the mean value of the relative TADG-15 mRNA expression ratio (TADG-15/*β*-tubulin) between groups. All *P*-values quoted are two-sided with statistical significance defined as a *P*-value <0.05. The Stat View 5 program (Abacus Concepts, Inc., Berkeley, CA, USA) was used for statistical analysis.

## RESULTS

### Immunohistochemical analysis of TADG-15

**Figure 1:**
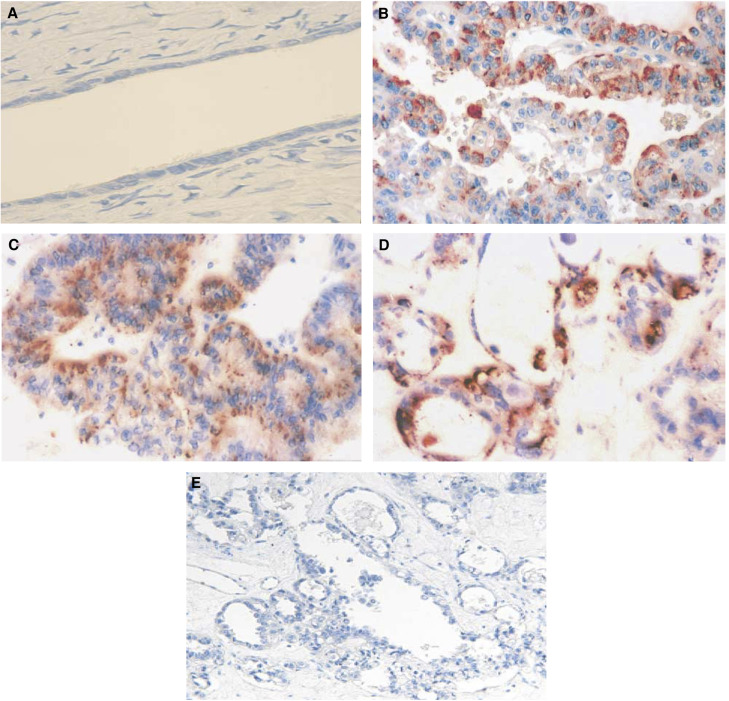
Immunohistochemistry. TADG-15 is absent in normal ovarian surface epithelial cells (**A**, × 100). Positive TADG-15 staining is observed in serous (**B**, × 100), endometrioid (**C**, × 100), and clear cell adenocarcinoma (**D**, × 100). Negative control section showed no nonspecific staining (**E**, same tumour as shown in **D**, × 50).

Immunohistochemical expression of TADG-15 was examined in 89 epithelial ovarian carcinomas and seven normal ovaries. Figure 1 shows representative samples. Positive TADG-15 staining was observed in both the cytoplasm and on the cell surface of ovarian carcinoma cells, whereas normal ovarian surface epithelium showed negative TADG-15 staining.

Of 89 ovarian carcinomas examined, 50 (56.2%) were positive for TADG-15. Table 1 shows positive TADG-15 expression in relation to each patient's age, clinical stage, histological type, and histological grade. TADG-15 was detected more frequently in stage I cases than in cases of advanced stage disease (stage I, 24 of 33: 72.7%; stage II/III/IV, 26 of 56: 46.4%; *P*=0.0157, *χ*^2^-test). In 33 stage I cancers, serous adenocarcinomas represent six cases, mucinous adenocarcinomas 12 cases, endometrioid adenocarcinomas six cases, and clear cell carcinomas nine cases. When expression was evaluated in relation to histological subtype, clear cell adenocarcinomas (14 of 14, 100%) expressed TADG-15 more frequently than serous (17 of 39, 43.6%), mucinous (10 of 19, 52.6%), or endometrioid adenocarcinomas (nine of 17, 52.9%). Out of 14 TADG-15 positive clear cell cancers, nine cases were stage I cancers. No significant correlation was found between positive TADG-15 expression and patient age or tumour histological grade.

### Survival analysis

**Figure 2:**
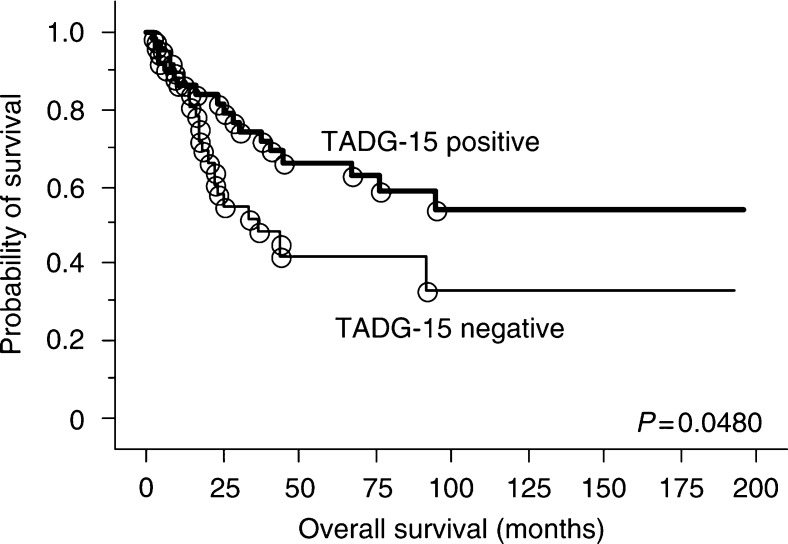
Log-rank testing shows that positive TADG-15 expression is significantly correlated with favourable overall survival in ovarian cancer patients (negative *vs* positive TADG-15 expression, *P*=0.0480).

Data from univariate and multivariate analysis on the influence of age, clinical stage, histological grade, and TADG-15 expression status on patient overall survival are summarised in Table 2. In univariate analysis, log-rank testing revealed that older age, advanced clinical stage, high histological grade, and negative TADG-15 expression were significantly correlated with poor patient overall survival (age, *P*=0.0008; stage, *P*<0.0001; grade, *P*=0.0043; TADG-15, *P*=0.0480). Ovarian cancer patients were categorised along a Kaplan–Meier survival curve according to negative *vs* positive expression of TADG-15, showing a statistically significant association between negative TADG-15 expression and poor patient overall survival (*P*=0.0480, log-rank test, Figure 2). In multivariate analysis, age (<50 years *vs* >51 years), clinical stage (stage I *vs* stage II/III/IV), histological grade (grade 1 *vs* grade 2/3), and TADG-15 expression status (positive *vs* negative) were selected as covariable. Clinical stage was identified as a significant and independent variable (*P*<0.0001), whereas none of the other variables were significantly associated with overall survival in multivariate models.

### Semiquantitative PCR analysis for TADG-15 mRNA expression

**Figure 3:**
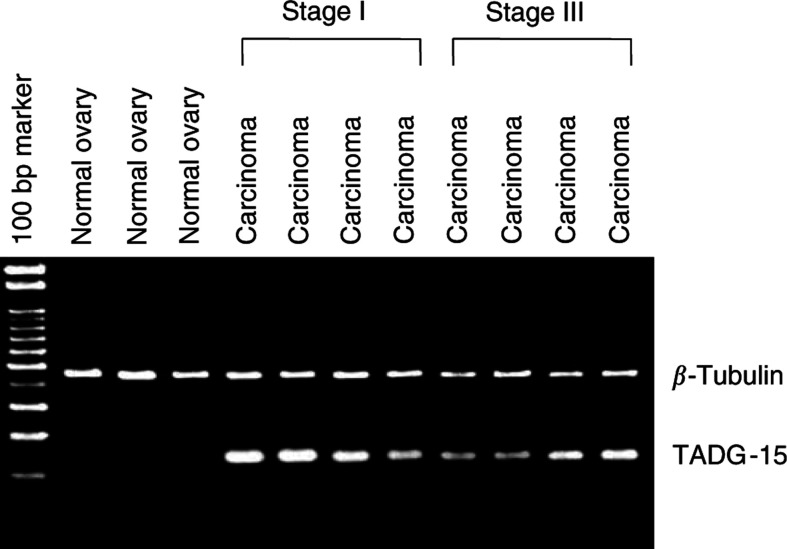
Semiquantitative PCR analysis of TADG-15 expression. Expression levels of TADG-15 relative to *β*-tubulin are significantly elevated in carcinomas compared with levels in normal ovaries.

In order to evaluate mRNA expression of TADG-15 in ovarian carcinomas, we performed semiquantitative PCR. In a preliminary study, the linearity of PCR amplification was confirmed. Figure 3 shows an example of semiquantitative PCR using TADG-15 primers coamplified with the internal control (*β*-tubulin) primers. Analysis of data as measured using the densitograph system and compared as ratios of expression (TADG-15/*β*-tubulin) indicated that TADG-15 expression was significantly elevated in carcinomas compared with that of normal ovaries (*P*<0.0001) (Table 3). For individual clinical stages of tumours, the mean value of relative TADG-15 mRNA expression ratio was 1.98±0.57 (mean±s.d.) for stage I tumours, 1.37±0.08 for stage II tumours, 1.64±0.34 for stage III tumours, and 1.69±0.31 for stage IV tumours, respectively. The TADG-15 mRNA expression ratio was significantly higher in stage I tumours than in stage II/III/IV tumours (*P*=0.0053) (Table 3).

## DISCUSSION

Recently, several studies have documented the enzymatic function of TADG-15 (Takeuchi *et al*, 1999, 2000; Lee *et al*, 2000; Friedrich *et al*, 2002). Analyses of the catalytic domain structure and the substrate specificity of TADG-15 (ST14/matriptase/MT-SP1) reveal that it has narrow substrate specificity; single-chain precursor of hepatocyte growth factor (HGF)/scattering factor (SF), protease-activated receptor 2 (PAR2) and single-chain urokinase plasminogen activator (uPA) are known macromolecular substrates of this protease (Lee *et al*, 2000; Takeuchi *et al*, 2000; Friedrich *et al*, 2002). These findings support the hypothesis that TADG-15 (ST14/matriptase/MT-SP1) is involved in regulatory events that require selective processing of particular substrates rather than nonselective degradation (Takeuchi *et al*, 1999). TADG-15 appears to play an important role in the development of cancer through its activation of some precursor proteins, of which active forms are crucial for cellular proliferation, cell motility, angiogenesis, and activation of other protease systems during tumour growth and invasion.

In the present study, we examined the expression of TADG-15 in ovarian cancer and its association with clinicopathological parameters and patient survival. Of 89 ovarian carcinomas, 50 (56.2%) produced high levels of TADG-15 that were detected by immunohistochemistry, whereas normal ovaries produced extremely low levels of this protein. This differential expression of TADG-15 between carcinoma and normal ovarian tissue was confirmed by semiquantitative PCR. Relative expression levels of TADG-15 mRNA from ovarian carcinomas were significantly higher compared with those of normal ovaries. This differential expression raises the possibility that TADG-15 might serve as a biomarker for ovarian cancer.

Among clinicopathological parameters we examined, clinical stage and histological type correlated with TADG-15 expression. With regard to histological type, clear cell adenocarcinoma showed TADG-15 expression more frequently than serous, mucinous, or endometrioid adenocarcinoma. One possible explanation of this difference among histological types is that there are alternative pathways of proteolytic reactions in tumour progression, and the pathway involving TADG-15 may correlate with some histological phenotypes including clear cell adenocarcinoma of the ovary. Oberst *et al* (2001) reported that TADG-15 expression was observed in tumours of epithelial origin but not in stromal-derived tumours and sarcomas. These findings support the hypothesis that there may be alternative pathways of proteolytic reactions correlating with histological type, cellular origin of malignancy, or both. With regard to clinical stage, TADG-15 expression was significantly more frequent in patients with stage I tumours than stage II/III/IV tumours. Furthermore, the mean value of relative TADG-15 mRNA expression ratio was also significantly higher in stage I tumours than in stage II/III/IV tumours. These findings indicate that increased expression of TADG-15 is mainly associated with early events in the development of malignancy (reflected as early stage disease) and its expression level is downregulated during cancer progression. This expression pattern suggests that TADG-15 may be important in establishing primary ovarian tumours and it later may function directly or indirectly as a progression inhibitor. Oberst *et al* (2002) investigated expression of TADG-15 (matriptase) and its inhibitor, hepatocyte growth factor activator inhibitor-1 (HAI-1), in ovarian cancer and reported that early stage tumours are more likely to express TADG-15 (matriptase) than advanced stage tumours and that TADG-15 (matriptase): HAI-1 ratio is increased in tumours of more advanced stage. Although we did not examine the expression of HAI-1 in the present study, our results also show that expression of TADG-15 is significantly associated with early stage ovarian cancer. Our results also demonstrate that patients with TADG-15-positive tumours have longer survival according to univariate analysis, although this statistical significance was lost in multivariate modeling. While TADG-15 might not be an independent prognostic factor for ovarian cancer, this protease is significantly associated with early stage disease and longer patient survival.

Previous studies have documented that some serine proteases are downregulated in various human carcinomas. For example, human kallikrein (KLK) 10 (normal epithelial cell specific-1; NES1) and KLK 14 are reported to be downregulated in breast cancer (Goyal *et al*, 1998; Yousef *et al*, 2001). Recently, Diamandis and Yousef (2002) reviewed human kallikreins, a subgroup of the serine protease family that colocalises at chromosomal region 19q13.4 and shares a high degree of homology. As described by these authors, some kallikreins can be useful biomarkers for poor prognosis of cancer, and others can be biomarkers for favourable prognosis. These findings indicate that proteolytic enzymes are not always associated with more aggressive forms of human cancer, even if they might be located at the same chromosomal region and share a high degree of homology. The process of tumour progression and invasion is thought to be a complicated phenomenon that involves a cascade of many kinds of proteases (Mignatti *et al*, 1986; Tryggvason *et al*, 1987; Liotta *et al*, 1991; Duffy, 1992). In this cascade, some proteases contribute to invasive tumour behaviour, and some proteases mediate activation of inhibitors of these proteases. Although the precise role of TADG-15 in this cascade remains unclear, our results demonstrated that increased expression of TADG-15 is frequent, especially in early stage ovarian cancer, which suggests that this enzyme may be involved in the initial spread of disease. TADG-15 therefore may serve as a marker for early detection of ovarian cancer.

## Figures and Tables

**Table 1:** Expression of TADG-15 in relationship to age, clinical stage, histological type, and histological grade in patients with ovarian carcinomas

	***N***	**TADG-15 positive for expression (%)**	***P*-value^a^**
Ovarian carcinoma	89	50 (56.2)	
*Patient age* (years)			0.2776
<50	40	25 (62.5)	
>51	49	25 (51.0)	
			
*Clinical stage*			0.0157*
Stage I	33	24 (72.7)	
Stage II/III/IV	56	26 (46.4)	
			
*Histological type*			0.0035*
Serous	39	17 (43.6)	
Mucinous	19	10 (52.6)	
Endometrioid	17	9 (52.9)	
Clear cell	14	14 (100)	
			
*Histological grade*			0.2442
Grade 1	54	33 (61.1)	
Grade 2/3	35	17 (48.6)	

a *χ*^2^-test.

* Statistically significant.

**Table 2:** Univariate and multivariate analysis on the influence of age, clinical stage, histological grade, and TADG-15 expression status on overall survival in patients with ovarian cancer

		**Multivariate^b^**		
**Factors**	**Univariate *P*-value^a^**	**RR^c^**	**95% CI^d^**	***P*-value**
Patient age (<50 years *vs* >51 years)	0.0008	0.525	0.246–1.121	0.0959
Clinical stage (stage I *vs* stage II/III/IV)	<0.0001	0.050	0.011–0.223	<0.0001
Histological grade (grade 1 *vs* grade 2/3)	0.0043	0.705	0.371–1.342	0.2872
TADG-15 expression (positive *vs* negative)	0.0480	1.097	0.577–2.087	0.7768

a Log-rank test.

b Cox proportional hazard model.

c RR, risk ratio.

d CI, 95% confidence interval.

**Table 3:** Relative expression levels of TADG-15 mRNA in normal ovaries and ovarian carcinomas

**Tissue type**	***N***	**TADG-15 (mean±s.d.)^a^**
Normal ovary	7	0.19±0.19^b^
*Ovarian carcinoma*	51	1.76±0.47^b^
Clinical stage of carcinoma		
Stage I	21	1.98±0.57^c^
Stage II/III/IV	30	1.61±0.32^c^

a s.d.=standard deviation.

b Normal *vs* carcinoma; *P*<0.0001.

c Stage I *vs* stage II/III/IV; *P*=0.0053.

All *P*-values were calculated using an unpaired *t*-test.
